# Atypical nontraumatic chylothorax in a monoclonal IgM elevated nodal marginal zone lymphoma: A case report and review of the literature

**DOI:** 10.3389/fimmu.2022.1031122

**Published:** 2022-10-27

**Authors:** Lingling Wang, Qian Huang, Jiao Tang, Jun Feng, Yongfen Huang, Jianming Dong, Yuexin Cheng, Hao Xu, Yuqing Miao

**Affiliations:** ^1^ Department of Hematology, The First People’s Hospital of Yancheng, The Yancheng Clinical College of Xuzhou Medical University, Yancheng, China; ^2^ Department of Oncology, The Second People’s Hospital of Huai’an, The Affiliated Huaian Hospital of Xuzhou Medical University, Huaian, China; ^3^ Department of Neurology, The First People’s Hospital of Yancheng, The Yancheng Clinical College of Xuzhou Medical University, Yancheng, China

**Keywords:** nodal marginal zone lymphoma, marginal zone lymphoma, chylothorax, B cell, immunotherapy, IgM, pleural effusion

## Abstract

Nodal Marginal Zone Lymphoma(NMZL) is an indolent lymphoma with a very low clinical incidence and is sometimes difficult to differentiate diagnostically from Lymphoplasmacytic lymphoma/Waldenstrom macroglobulinemia (LPL/WM). NMZL with elevated monoclonal immunoglobulin M (IgM) is even rarer. Nontraumatic chylothorax can be seen in aggressive lymphoma, which often happens with chest tightness and dyspnea as the primary clinical manifestation. We reported the first case of monoclonal IgM elevated NMZL complicated by atypical nontraumatic chylothorax. A 64-year-old male patient was first admitted to the Department of Respiratory Medicine with symptoms of chest tightness and shortness of breath. He was given several times thoracentesis to drain pleural effusion to improve pulmonary compression symptoms. The patient had a combination of elevated monoclonal IgM and atypical lymph node biopsy pathology. After two times lymph node biopsies and genetic testing, the patient was finally diagnosed with NMZL. Within a short time, he was admitted to the Department of Hematology due to the reappearance of massive pleural effusion, which indicated chylothorax. The patient repeatedly presented with left-sided pleural effusion, and the color went from red to yellow, and finally white. Only about half of the chylothorax cases present with typical clinical manifestations. We report this case intending to draw the attention of clinicians to hematologic malignancies with atypical nontraumatic chylothorax.

## Background

Marginal zone lymphoma(MZL) is a group of B-cell lymphomas that originate from the marginal zone of the lymph node and can occur in the spleen, lymph nodes, and mucosal lymphoid tissue, accounting for approximately 10% of all non-Hodgkin’s lymphomas (NHL). The incidence of MZL is low, ranging from approximately 5%-15% in Western countries to 7.8% in China. MZL is highly heterogeneous and is classified by the WHO into three types: extranodal marginal zone lymphoma (EMZL), NMZL, and splenic marginal zone lymphoma (SMZL). EMZL is the most common type, accounting for approximately 70% of all MZL, and has a relatively good prognosis. SMZL accounts for approximately 20% of all MZL, while NMZL is the rarest type of MZL (1). NMZL is very rare, so the clinical presentation is based mainly on reported case data and a small number of patients. Most patients will present with scattered peripheral, abdominal and thoracic lymph node invasion and are usually not of the giant type. At the time of diagnosis, extranodal and splenic involvement need to be excluded so that it can be differentiated from other types of MZL. The median age at diagnosis is 50-64 years old and the patient is usually in the good physical condition and free of B symptoms. Bone marrow involvement occurs in about 1/3 of patients, and peripheral blood involvement is rarer (2).

Disrupted or dysfunctional chyle flow in the thoracic duct can cause chylothorax. Therefore, the etiology of chylothorax can be broadly classified as nontraumatic or traumatic (medically oriented or blunt contusion or penetrating injury). The etiology of chylothorax may vary depending on the patient population admitted to the medical institution. However, malignancy is often the primary cause of nontraumatic chylothorax, whereas thoracic surgery is the primary cause of traumatic chylothorax (3). Retrospective clinical studies found equal proportions of traumatic and nontraumatic etiologies.

Herein, we reported our experience with the first case of monoclonal IgM elevated NMZL complicated by atypical nontraumatic chylothorax. The patient was first admitted to the Department of Respiratory Medicine with symptoms of chest tightness and shortness of breath. He was in critical condition and was given several times thoracentesis to drain pleural effusion to improve pulmonary compression symptoms. The color went from red to yellow, and finally white. A lymph node biopsy was performed to clarify the pathology. The patient had a combination of elevated monoclonal IgM and atypical pathology and was finally diagnosed with NMZL. Within a short time, he was admitted to the hospital several times due to the recurrence of massive pleural effusion. The patient was allergic to multiple drugs while on the first-line chemotherapy regimen and had quite acute side effects. The pleural effusion recurred and was eventually completely absorbed with the second-line GB chemotherapy regimen for four cycles. The patient did not undergo drug-related allergic reactions again. The patient eventually achieved CR and is currently in clinical follow-up.

## Case presentation

In June 2021, a 64-year-old male patient was admitted to the Department of Respiratory Medicine of our hospital and complained of chest tightness and shortness of breath for more than 20 days. The chest CT showed that lymph nodes were partially enlarged in the right lower lung hilar, bilateral axillar, and retroperitoneal. In addition, there was a large amount of effusion in the left pleural cavity. Physical examination revealed multiple superficial lymph node enlargements throughout the body, breath sounds could not be heard on the left side, and did not reveal hepatomegaly or splenomegaly. Thoracentesis and drainage were performed in the Department of Respiratory Medicine. The patient’s chest tightness and shortness of breath improved significantly. The tuberculosis test was negative. The hemoglobin level was decreased at 95g/L, leukocytes at 8.84×10^9^/L, and platelets at 258×10^9^/L. The color of the pleural effusion was red. A laboratory examination of the pleural effusion was as follows, monocytes at 96%, nucleated cell count at 2100×10^6^/L, erythrocytes at 40-50/HF, ADA at 11.4U/L, glucose at 8.26mmol/L, and total protein at 47.8g/L. Biochemical showed an elevated globulin at 62.4g/L, albumin at 27.2g/L, lactate dehydrogenase(LDH) at 147.2U/L, β2 microglobulin at 4mg/L, and an elevated monoclonal IgM at 55.6g/L. A right supraclavicular lymph node biopsy was performed on day 5 of hospitalization. Combined with immunochemical staining and HE staining, histopathological revealed that this case is a B-cell lymphoma. Immunohistochemical results were as follows: LCA (+), CD20 (+), S100 (-), actin (-), SMA (-), Ki-67 (20% +), HMB45 (-), CD31 (+), κ (plasma cell +), λ (-), CD79α (+), CD5 (-), CD3 (background cell +), CD10 (-), CD21 (FDC +), CD23 (FDC+), CD38 (+), CD138 (+), CD43 (+), CyclinD1 (-), Bcl-2 (+), Bcl-6 (30% +), MUM1 (+), PAX5 (+), c-Myc (-), CD19 (+). Immunophenotyping was incomplete, and genetic testing was not done. Due to doubts about the immunohistochemical results and difficulties in distinguishing which subtype of B-cell lymphoma, the patient went to Ruijin Hospital, Shanghai Jiaotong University School of Medicine for an axillary lymph node biopsy in August 2021. An immunohistochemistry analysis were as follows: CD20 (+), CD79a (+), CD19 (+), CD22 (+), BCL-2 (+), IgD (+), CD23 (+), Ki67 (2% +), CD37 (50% +), CD10 (-), BCL-6 (-), C-myc (-), MUM-1 (-), Kappa (-), Lambda (-), CD30 (-), CD38 (-), CD3 (-), CD5 (-), CyclinD1 (-), PD-1 (-), CD138 (-), CD21 (+). Epstein-Barr virus-encoded small RNA *in situ* hybridization (EBER-ISH) was negative. The somatic mutation of MYD88L265P and CXCR4 were negative. Combined with the patient’s lymph node biopsies immunohistochemistry and clinical presentation, the patient was finally diagnosed with monoclonal IgM elevated NMZL. But the patient refused chemotherapy for personal reasons. In September 2021, the patient was transferred to the local hospital with a recurrence of chest tightness and shortness of breath. CT examination suggested a large amount of effusion in the left pleural cavity, and thoracentesis and drainage were performed again.

In October 2021, the patient was transferred to our hospital for further treatment. Flow cytometry of bone marrow showed that lymphocytes occupied about 12.7% of nuclear cells, of which CD19+ cells occupied about 6.2% of nuclear cells, expressing HLA-DR, CD19, CD20, cKappa, FMC-7, and CD38, and negative for CD5, CD10. There were no abnormalities in the chromosome examination. Referring to the 2014 Lugano staging, this patient was in stage IV group B. The patient chose chemotherapy of rituximab and lenalidomide. The patient developed a widespread rash with pruritus after taking lenalidomide and was considered to be allergic to it, which improved after discontinuation of the drug and anti-allergic treatment.

The patient developed shortness of breath and facial swelling during hospitalization, and the ultrasound examination of the chest cavity indicated a large amount of pleural effusion on the left side again. Thoracentesis drainage was performed for the third time, and the the color of pleural effusion was yellow. A flow cytometric analysis of the pleural effusion indicated abnormal monoclonal B cells, which expressed HLA-DR, CD19, CD20, CD22, cKappa, and were negative for CD5, CD10, and FMC-7. The flow cytometry indicated that pleural effusion may be associated with invasion of the pleura. Because of the patient’s allergy to lenalidomide, the patient received the BR (rituximab 600mg d1, bendamustine 150mg d2-3) chemotherapy regimen in November and December separately. The patient underwent chills, vomiting, and diarrhea during rituximab treatment and improved with symptomatic supportive treatment.

**Figure 1:**
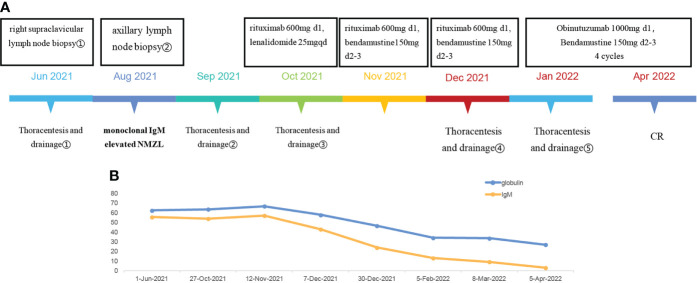
**(A)** The treatment timeline of this patient. **(B)** The decreasing trend of globulin and IgM in this patient during the inpatient and outpatient follow-up period from June 2021 to April 2022.

**Figure 2:**
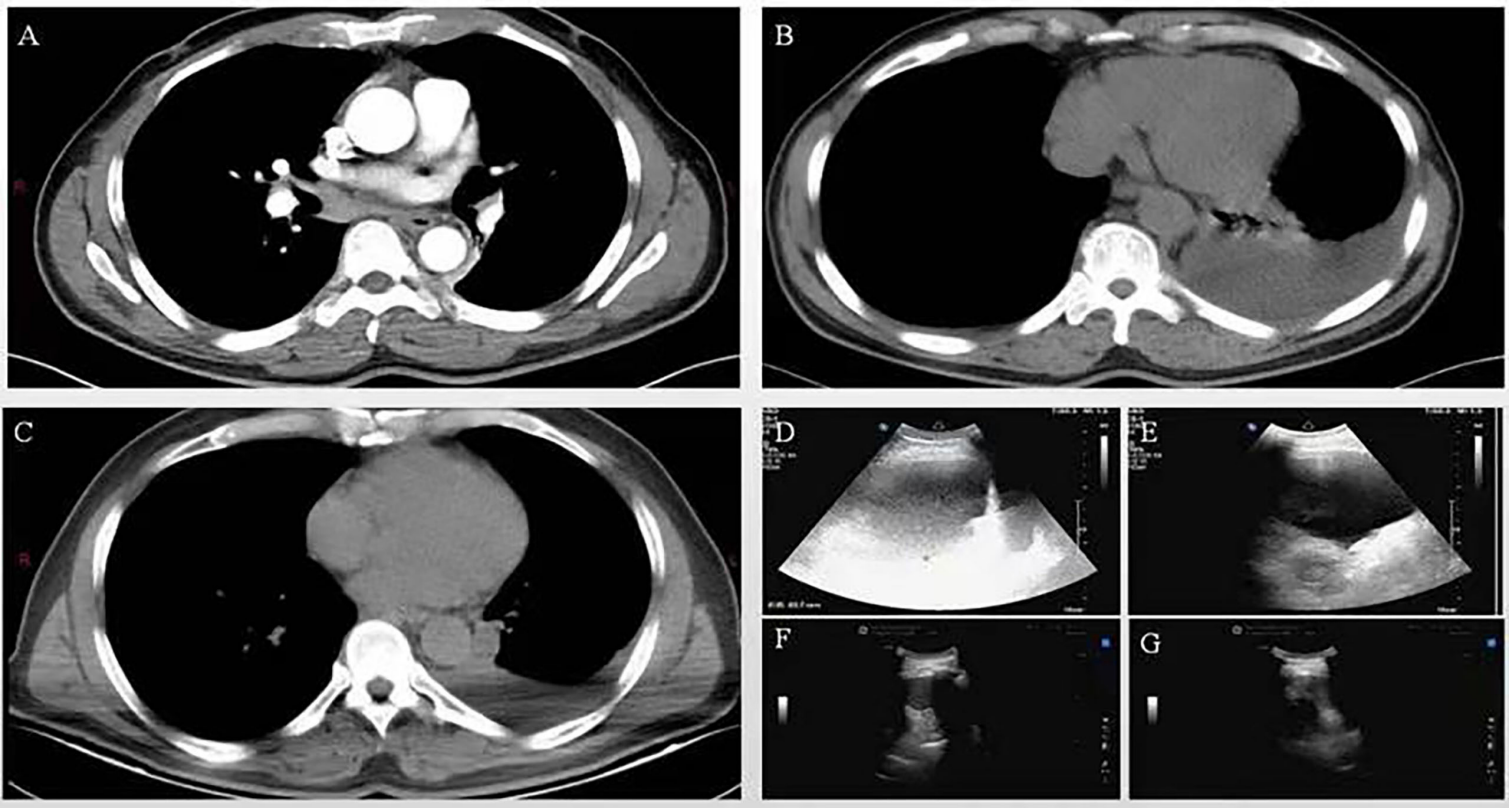
**(A)** In June 2021, CT suggested enlarged lymph nodes in the right hilar lung. **(B, C)** In February and March 2022, CT suggested a gradual decrease in pleural effusion. **(D, E)** In June 2021, a chest ultrasound suggested a large pleural effusion. **(F, G)** No pleural effusion was detected by chest ultrasound in April 2022.

Shortly after the second BR chemotherapy treatment, the patient was readmitted to our hospital with chest tightness and shortness of breath. Ultrasound examination showed a large pleural effusion on the left side, and ultrasound-guided thoracentesis was performed for the fourth time to drain the cavity. The pleural effusion color was milky white, and the Levantine test showed positive. 84% were mononuclear cells, and the nucleated cell count was up to 645×10^6^/L. The level of total protein was at 49.5 g/L, LDH at 110.2 U/L, and glucose at 7.25 mmol/L. The chyle test of pleural effusion was positive. The somatic mutation of MYD88L265P of pleural effusion was negative. Immunophenotyping of the pleural effusion suggested that lymphocytes occupied about 71.3% of the nucleated cells, with CD19+ cells occupying about 1% of the nucleated cells, expressing HLA-DR, CD19, CD20, CD22, and Kappa. Considering that the patient developed a severe allergic reaction to rituximab, we adjusted to GB (Obinutuzumab 1000mg d1, Bendamustine 150mg d2-3) chemotherapy regimen in January 2022. One month later, the patient’s chest ultrasound showed a significant reduction of pleural effusion in the left side of the chest cavity. The patient received another two cycles of treatment. In April 2022, pleural effusion could not be detected by ultrasound. The patient performed GB chemotherapy for the fourth course. The patient did not experience drug-related allergies or reignition of pleural effusion. Most importantly, the globulin and IgM gradually decreased to normal. The patient is currently in follow-up and maintaining remission. The patient is currently on maintenance therapy with the GB regimen. No further pleural effusion was observed on repeat chest CT (**Figure 1A**. The treatment timeline of this patient. **Figure 1B** The decreasing trend of globulin and IgM in this patient during the inpatient and outpatient follow-up period from June 2021 to April 2022. **Figure 2A** In June 2021, CT suggested enlarged lymph nodes in the right hilar lung. **Figures 2B, C** In February and March 2022, CT suggested a gradual decrease in pleural effusion. **Figures 2D, E** In June 2021, a chest ultrasound suggested a large pleural effusion. **Figures 2F, G** No pleural effusion was detected by chest ultrasound in April 2022).

## Discussion

**Figure 3:**
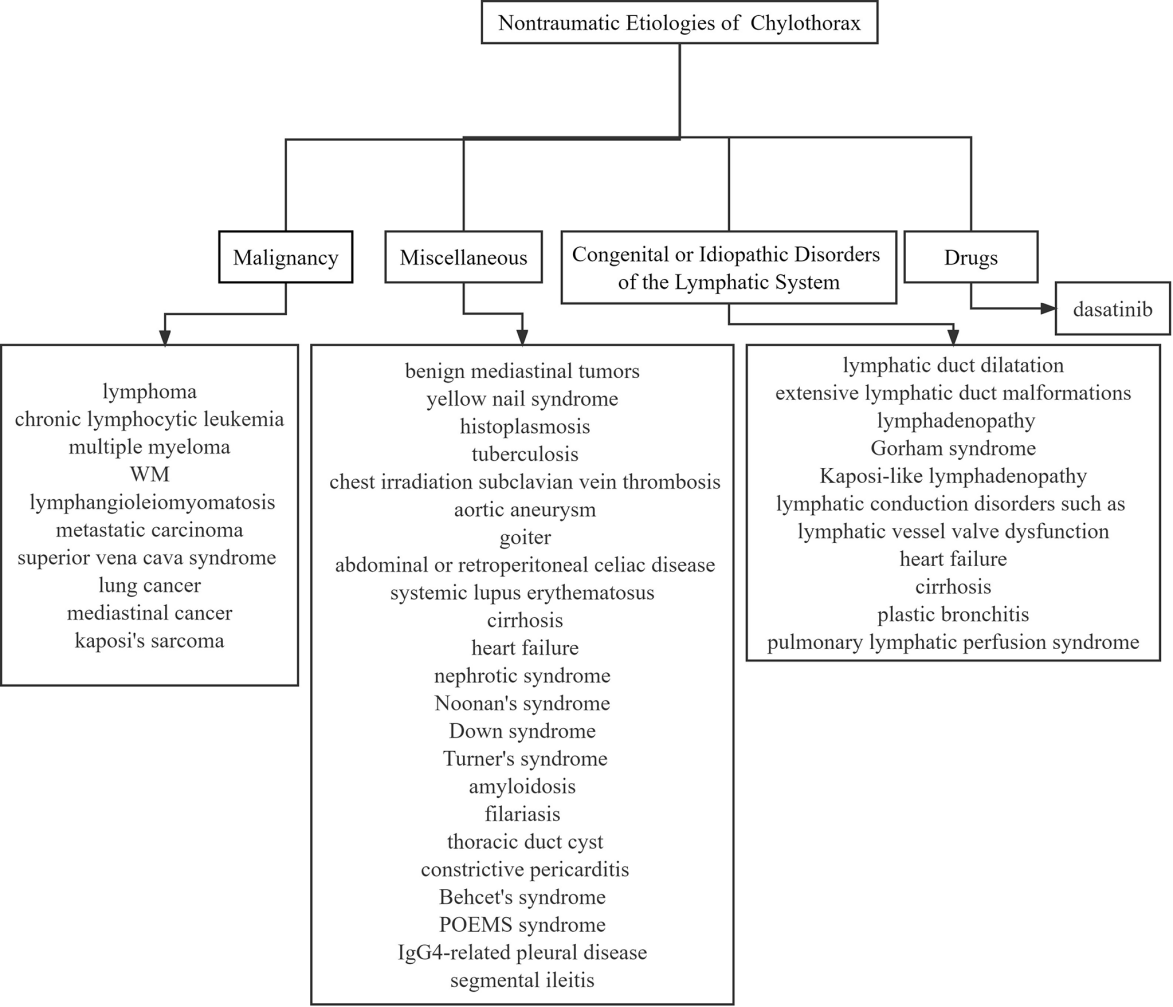
Nontraumatic etiologies of chylothorax.

NMZL has a very low incidence rate, which is indolent and rarely combined with B symptoms. In some cases, it is difficult to differentiate from other B-cell lymphomas (4). The prognosis of NMZL with aggressive onset is generally poor, and there are no reports of NMZL in combination with chylothorax. Herein, our team reports a case of monoclonal IgM elevated NMZL with recurrent chylothorax. Most importantly, the patient’s chylothorax was atypical. The color went from red to yellow, and finally white. We also summarize the pathogenesis (**Table 1**) (5–8), and nontraumatic etiologies of chylothorax (**Figure 3**).

**Table 1:** The Pathogenesis of Chylothorax.

Abnormalities of the thoracic ducts or lymphatic vessels	
**Compressive Obstruction**	**malignant tumor**
Direct Involvement	malignant or infectious lymphadenitis
	surgically caused lacerations or ruptures
	blunt contusions
	penetrating injuries
Malformations	Gorham syndrome
	extensive lymphatic duct malformations
	lymphangioleiomyomatosis
Dysfunction	celiac reflux into the lungs
Irritation by Excess Lymphatic Effusion	lymphatic vessel rupture
	celiac leakage
Abnormal Transport Mechanism	celiac transit through the diaphragm from an accumulation in the peritoneal or retroperitoneal cavity

This patient had a markedly elevated IgM at the initial diagnosis, making it difficult to differentiate from LPL/WM. Monoclonal IgM elevation with clinical symptoms is most commonly seen in LPL/WM, but a very small percentage of MZL can also show monoclonal IgM elevation. The typical immunophenotypes of MZL are CD5-, CD10-, CD20+, and CD23-/+, often with chromosomal abnormalities such as t (11;18), t (14;18), t (3;14), and t (1;14) (9). However, it is similar to LPL/WM in terms of clinical and pathological features, and sometimes it is difficult to make a differential diagnosis. The somatic mutation of MYD88L265P is present in about 90% of LPL/WM patients, while the incidence of this mutation is only 7% in SMZL and MALT lymphomas, and it is rare in NMZL (10). This suggests that MYD88L265P can be used for differential diagnosis.

This patient repeatedly presented with pleural effusion, and the color of the pleural effusion went from red to yellow, and finally white. In patients with persistent or recurrent pleural effusions of unknown etiology, chylothorax should be considered if a pleural cavity aspiration or chest drainage reveals a milky, cloudy or bloody. Only about half of patients with chylothorax have a milky or milky white pleural effusion, so physicians need to be on high alert, especially in patients with a history of chest, neck, or abdominal surgery (11). Pleural effusion may become milky after ingestion of high-fat foods. Chylothorax could be detected on chest X-Ray and CT. About 78% of patients have unilateral chylothorax, with 67% having right-sided pleural involvement and 33% having left-sided pleural involvement. However, bilateral effusions may also occur. The anatomical site of thoracic duct injury or obstruction can affect the location of the effusion. The thoracic duct crosses the mediastinum from right to left at the level of the fifth thoracic vertebra, and injury or obstruction of the thoracic duct below this level usually results in a right-sided chylothorax. Conversely, injury or obstruction of the thoracic duct above this level often results in a left-sided chylothorax. Lesions at any level of the mediastinum that disrupt the lymphatic reticular anastomosis and branches of the thoracic duct may produce chylothorax on either or both sides.

The patient underwent a second lymph node biopsy in August 2021, and pathological examination did not reveal diffuse growth of large B cells. After he was transferred to our center in October 2021, he underwent a bone marrow biopsy and re-evaluated his condition. Genetic examination of the patient did not detect TP53 mutation, NOTCH1 mutation, CDKN2A/B deletion, Bcl-2 overexpression, or MYC abnormal expression. Therefore, the transformation of NMZL into diffuse large B cell lymphoma(DLBCL) can be ruled out. However, close follow-up is still needed. The possibility of this kind of transformation should be vigilant if clinical symptoms such as enlargement of lymph nodes, cytopenia, B symptoms, elevated LDH, hypercalcemia, or new genetic abnormalities occur. New small molecules that modulate the pathways involved in the molecular pathogenesis of B-cell lymphoma, monospecific and bispecific monoclonal antibodies, drug-immunoconjugates, and cellular therapies are novel therapeutic options that are available for B-cell lymphoma (12). The final choice of GB regimen chemotherapy for this patient was primarily based on a global multicenter randomized controlled phase III clinical study called GADOLIN. For many patients receiving rituximab who have failed treatment, the new combination of obinutuzumab plus bendamustine will greatly improve progression-free survival(PFS) and overall survival(OS) (13). Considering this patient was allergic to numerous first-line drugs, we chose GB chemotherapy, which proved to be effective.

Patients with chylothorax tend to have difficulty breathing due to the mechanical compression of the pleural effusion. Other symptoms include feelings of heaviness in the chest, weakness, and weight loss (14). Fever and chest pain are rare because the chylous effusion in the pleural cavity does not cause an inflammatory response and patients are rarely infected due to the antibacterial effect of immunoglobulins in the chylous effusions. Coughing up chylous effusions is an extremely rare manifestation of chylothorax. Because chyle contains protein and fat, patients may present with malnutrition or fat-soluble vitamin deficiency, but this is more likely to be related to the clearance of large amounts of chyle rather than to the accumulation of chyle in the pleural cavity, where these components can be slowly absorbed. Similarly, because chronic chyle loss may cause immunosuppression associated with immunoglobulin loss, patients may be susceptible to organ infections outside the pleural cavity. Physical examination may reveal diminished breath sounds as well as turbid sounds on percussion, depending on the amount and location of the effusion collection. Nontraumatic chylothorax (e.g., malignancy) usually develops gradually over several weeks, whereas post-traumatic or post-operative chylothorax may develop very rapidly if the amount of leakage is large (>500 mL/d) or may occur within days (2-10 days) after trauma due to a slow accumulation of leaking chyle.

NMZL is a clinically rare subtype of MZL, and there are no previous clinical reports of NMZL combined with chylothorax. To the best of our knowledge, this is the first clinical report of monoclonal IgM elevated NMZL combined with chylothorax. A retrospective study has analyzed the clinical features of NHL combined with pleural effusion. A total of 430 patients were included in this study. The mean age was 58 years, 64% of the patients were male, 50% of the patients had pleural effusion at the time of NHL diagnosis, 62.5% of the pleural effusions occurred unilaterally, and 90% were exudate (15). There are many reports of pleural effusion as the first clinical manifestation with a final diagnosis of lymphoma, but very few of chylothorax onset. This patient presented with massive pleural effusion as the first clinical manifestation, accompanied by generalized lymph node enlargement. The color went from red to yellow, and finally white, which indicated atypical chylothorax. The mechanisms by which chylothorax occurs in NMZL may include the following reasons. First, compressional obstruction leads to abnormalities in the thoracic duct or lymphatic vessels. Second, direct invasion by lymphoma. These cause disruption or dysfunction of chyle flow in the thoracic duct, which leads to the development of chylothorax in this patient.

## Conclusions

NMZL is a clinically rare subtype of MZL, and there are no previous clinical reports of monoclonal IgM elevated NMZL combined with atypical chylothorax. The patient repeatedly presented with left-sided pleural effusion, and the color went from red to yellow, and finally white. Only about half of the chylothorax cases present with typical clinical manifestations. We report this case intending to draw the attention of clinicians to hematologic malignancies with atypical chylothorax.

## Data availability statement

The original contributions presented in the study are included in the article/supplementary material. Further inquiries can be directed to the corresponding author.

## Ethics statement

The studies involving human participants were reviewed and approved by The First People’s Hospital of Yancheng. The patients/participants provided their written informed consent to participate in this study. Written informed consent was obtained from the individual(s) for the publication of any potentially identifiable images or data included in this article.

## Author contributions

LW and QH conceived and designed the study, they contributed equally to this work and share the first authorship. JT, JF, YH, JD, YC, HX collected the clinical data. LW and YM wrote the paper. All authors contributed to the article and approved the submitted version.

## Funding

This work was supported by Yancheng Municipal Health Commission (Grant Number YK2020004).

## Conflict of interest

The authors declare that the research was conducted in the absence of any commercial or financial relationships that could be construed as a potential conflict of interest.

## Publisher’s note

All claims expressed in this article are solely those of the authors and do not necessarily represent those of their affiliated organizations, or those of the publisher, the editors and the reviewers. Any product that may be evaluated in this article, or claim that may be made by its manufacturer, is not guaranteed or endorsed by the publisher.
